# Functional Variation in the *FAAH* Gene Is Directly Associated with Subjective Well-Being and Indirectly Associated with Problematic Alcohol Use

**DOI:** 10.3390/genes14091826

**Published:** 2023-09-21

**Authors:** Lisa Bornscheuer, Andreas Lundin, Yvonne Forsell, Catharina Lavebratt, Philippe A. Melas

**Affiliations:** 1 Department of Public Health Sciences, Stockholm University, 10691 Stockholm, Sweden; lisa.bornscheuer@su.se; 2 Department of Global Public Health, Karolinska Institutet, 17177 Stockholm, Sweden; andreas.lundin@ki.se (A.L.); yvonne.forsell@ki.se (Y.F.); 3 Department of Molecular Medicine and Surgery, Karolinska Institutet, 17176 Stockholm, Sweden; catharina.lavebratt@ki.se; 4 Center for Molecular Medicine, L8:00, Karolinska University Hospital, 17176 Stockholm, Sweden; 5 Center for Psychiatry Research, Department of Clinical Neuroscience, Karolinska Institutet & Stockholm Health Care Services, 11364 Stockholm, Sweden

**Keywords:** fatty acid amide hydrolase, rs324420, Pro129Thr, anandamide, endocannabinoid system, happiness, alcoholism

## Abstract

Fatty acid amide hydrolase (FAAH) is an enzyme that degrades anandamide, an endocannabinoid that modulates mesolimbic dopamine release and, consequently, influences states of well-being. Despite these known interactions, the specific role of FAAH in subjective well-being remains underexplored. Since well-being is a dynamic trait that can fluctuate over time, we hypothesized that we could provide deeper insights into the link between FAAH and well-being using longitudinal data. To this end, we analyzed well-being data collected three years apart using the WHO (Ten) Well-Being Index and genotyped a functional polymorphism in the *FAAH* gene (rs324420, Pro129Thr) in a sample of 2822 individuals. We found that the A-allele of rs324420, which results in reduced FAAH activity and elevated anandamide levels, was associated with lower well-being scores at both time points (Wave I, B: −0.52, *p* = 0.007; Wave II, B: −0.41, *p* = 0.03, adjusted for age and sex). A subsequent phenome-wide association study (PheWAS) affirmed our well-being findings in the UK Biobank (N = 126,132, alternative C-allele associated with elevated happiness, *p* = 0.008) and revealed an additional association with alcohol dependence. In our cohort, using lagged longitudinal mediation analyses, we uncovered evidence of an indirect association between rs324420 and problematic alcohol use (AUDIT-P) through the pathway of lower well-being (indirect effect Boot: 0.015, 95% CI [0.003, 0.030], adjusted for AUDIT in Wave I). We propose that chronically elevated anandamide levels might influence disruptions in the endocannabinoid system—a biological contributor to well-being—which could, in turn, contribute to increased alcohol intake, though multiple factors may be at play. Further genetic studies and mediation analyses are needed to validate and extend these findings.

## 1. Introduction

Subjective well-being, although complex to define, is a critical construct in understanding human behavior and health. It is typically characterized as a positive evaluation of one’s life and a general sense of contentment [1]. While well-being is significantly influenced by societal and social factors, such as interpersonal relationships, housing, employment, and institutional trust [2,3], there is also evidence supporting a role for specific genes. On average, heritability estimates for various well-being constructs are about 42% [4]. So far, however, single nucleotide polymorphisms (SNPs) account only for roughly 6.3% of this heritability [5,6,7]. The complexity in defining and measuring well-being, combined with its moderate heritability estimates, likely contributes to the difficulties in elucidating its underlying genetic architecture. Therefore, insights from preclinical and translational research can provide valuable guidance for studies aiming to identify key genes influencing well-being.

The human *FAAH* gene encodes for the enzyme fatty acid amide hydrolase (FAAH), which degrades N-arachidonoyl ethanolamine (AEA), commonly known as anandamide, a high-affinity partial agonist for the cannabinoid receptor 1 [8,9]. Anandamide is known to modulate mesolimbic dopamine release [10], a critical component of the reward system involved in the perception of pleasurable experiences [11] and subjective well-being [12,13]. However, the neurobiological effects of anandamide are complex and do not fully mirror those produced by exogenous substances like cannabis [14]. For instance, pharmacogenetic studies using either FAAH inhibitors or *Faah* knockout mice have demonstrated that increased anandamide levels can modulate emotional states without inducing the typical symptoms of cannabinoid intoxication, such as reduced body temperature, catalepsy, or heightened feeding behavior [9]. Moreover, stress has been found to reduce anandamide levels [15] and pharmacogenetic studies suggest that FAAH inhibition could help regulate stress-related and affective behaviors [16,17,18,19,20].

Despite the recognized relationship between anandamide and well-being, only one previous study investigated the link between the *FAAH* gene and well-being using allele frequency database data [21]. Moreover, no research to date has examined this link within a longitudinally assessed human population. Longitudinal studies offer a unique advantage over cross-sectional studies by allowing researchers to observe changes within individuals over time. This design can identify temporal relationships and causality, making it particularly apt for studying dynamic traits like subjective well-being, which can fluctuate over time. In response to this gap, we utilized data from a population-based cohort assessed at two distinct time points, three years apart, and genotyped a functional genetic variant in the *FAAH* gene, rs324420. This SNP, often referred to as the Pro129Thr variant, results in an amino acid substitution, with the A-allele leading to a FAAH variant with reduced expression and activity which increases anandamide levels [22,23,24]. Additionally, rs324420 has a RegulomeDB rank of 1f and a score of 0.66703, suggesting a high likelihood that this variant affects transcription factor binding and is linked to changes in gene expression. Building on these findings, the present study aims to study the relationship between rs324420 and subjective well-being, with an added focus on its potential link with problematic alcohol use inspired by our PheWAS findings.

## 2. Methods

### 2.1. Participants

PART (an acronym from the Swedish ‘Psykisk hälsa, Arbete och RelaTioner’), is a longitudinal cohort project designed to investigate the risk and protective factors for mental health in Stockholm County, Sweden [25]. The current study utilized rs324420 genotyping data and information extracted from self-administered questionnaires in PART Wave I (1998–2000; 53% response rate, N = 10,443) and PART Wave II (2001–2003; 84% response rate, N = 8613) [26,27]. These questionnaires encompassed areas such as demographics, subjective well-being, mental health problems, social support, stressful life events, childhood adversities, and alcohol use, detailed below. The PART study has not conducted a specific test for population stratification. However, it is noteworthy to mention that the cohort is predominantly Swedish, with only 11% of the participants being of non-Swedish origin. Within this subset, the overwhelming majority are of Nordic descent, primarily Finnish [25]. The PART project was conducted in accordance with the Code of Ethics of the World Medical Association’s (WMA) Declaration of Helsinki and approved by the ethical review board at Karolinska Institutet. All participants gave informed consent.

### 2.2. Subjective Well-Being

Subjective well-being, often defined as a person’s cognitive and affective evaluations of their life [28], was assessed using the WHO (Ten) Well-Being Index [29], a derivative of the WHO (Bradley) Subjective Well-Being Inventory Index [30]. This scale comprises ten items, with a recall period of the previous week. Six items pertain to coping skills and life adjustment (i.e., cognitive evaluations), while the remaining four items focus on symptoms of depression, anxiety, and vitality (i.e., affective evaluations). These items were scored on a scale from ‘never’ (0) to ‘always’ (3). Higher scores indicate greater well-being, with total scores ranging from 0 to 30. Subjective well-being scores were calculated separately for PART Waves I and II.

### 2.3. Depression and Anxiety Diagnoses

Depression diagnoses, including major depression, mixed anxiety depression, or dysthymia as per DSM-IV, were identified using the Major Depression Inventory (MDI) supplemented with questions regarding disability due to psychological symptoms [31,32]. These assessment items corresponded to the two weeks preceding questionnaire completion. Anxiety diagnoses, consistent with DSM-IV, were determined using the Sheehan Patient Rated Anxiety Scale [33] and the phobia/avoidance component of an instrument by Marks & Mathews [34]. Even though the American Psychological Association (APA) now classifies obsessive–compulsive disorders (OCD) as a distinct mental health condition, we incorporated it into the anxiety group using screening questions recommended by the Swedish Psychiatric Association and the Swedish Institute for Health Services Development [35]. This was carried out to maintain consistency with previous PART publications relying on DSM-IV criteria for an anxiety diagnosis. A history of depression or anxiety was attributed to individuals if symptoms were reported in either PART Wave I or II.

### 2.4. Stressful Life Events and Childhood Adversities

Data from PART Waves I and II concerning stressful life events and childhood adversities were extracted and analyzed as previously described [36,37,38,39,40]. In brief, stressful life events referred to incidents occurring within 12 months before questionnaire completion, scored based on 28 stressful items including, but not limited to, interpersonal conflicts, separation, severe illness or death of a loved one, significant work-related issues, abortion, and family member victimization. Childhood adversities, occurring before the age of 18, encompassed loss of a parent, parental divorce, serious financial hardships, and severe family conflicts. Both stressful life events and childhood adversities were treated as categorical variables (none or at least 1). Childhood adversity data were obtained from the PART Wave I questionnaire, while stressful life event data were retrieved and separately analyzed for PART Waves I and II.

### 2.5. Social Support

The PART questionnaires include two items specifically assessing social support: (i) “There is one person in particular that I feel I can really get support from”, and (ii) “Apart from those at home, there are others I can turn to when I’m having a hard time, someone I can easily meet, that I trust and can get real help from when I’m in trouble”. Both items were scored on a four-point scale, ranging from “true” to “not true at all”. As these items showed strong correlation (Spearman’s rho: 0.477, *p* < 0.001 for PART Wave I and Spearman’s rho: 0.492, *p* < 0.001 for PART Wave II), we consolidated them into a single social support variable, categorized as high (i.e., scoring at or above the mean of both questions) or low (i.e., scoring below the mean). Social support was calculated and analyzed separately for PART Waves I and II.

### 2.6. Alcohol Consumption and Problematic Alcohol Use

The Alcohol Use Disorders Identification Test (AUDIT) was utilized to examine alcohol consumption and alcohol-related problems. This tool, previously validated in the PART study [41], comprises 10 items with a total score ranging from 0–40, reflecting drinking habits and associated problems over the past 12 months. AUDIT data from PART Waves I and II were extracted and analyzed in terms of the complete AUDIT scale (items 1–10) and the AUDIT-P scale (items 4–10) that assesses problematic alcohol use.

### 2.7. DNA Collection and Genotyping

DNA samples were collected from a subset of participants (N = 3018) who responded to PART Waves I and II, using self-administered saliva collection kits (Oragene DNA sample collection kit; DNA Genotek Inc., Stittsville, ON, Canada) as described previously [42,43]. For this study, genotyping of rs324420 was conducted in N = 2915 individuals using a TaqMan SNP genotyping assay on an ABI 7900 HT instrument (Thermo Fisher Scientific, Waltham, MA, USA). N = 93 genotyping reactions (3.19%) were ambiguous and were thus excluded from subsequent analyses. We assessed genotyping quality by testing for deviation from the Hardy–Weinberg equilibrium, setting *p* at <0.05.

### 2.8. Phenome-Wide Association Study

We employed the Atlas of GWAS Summary Statistics (GWAS Atlas, https://atlas.ctglab.nl/, last accessed on 9 August 2023) [44] to conduct a phenome-wide association study (PheWAS) for rs324420, aiming to validate our findings in distinct cohorts. We set the maximum *p*-value threshold at 0.01 for this analysis.

### 2.9. Statistical Analyses

Normality of the data was assessed using the Shapiro–Wilk test and homoscedasticity was checked with Levene’s test. Spearman’s rank correlation was used to measure the correlation between ordinal variables. Differences in subjective well-being scores across groups defined by age, sex, education, social support, mental health diagnosis, stressful life events, and childhood adversities were assessed using the Kruskal–Wallis test. We constructed linear regression models to probe the relationships between rs324420 genotypes, subjective well-being, and AUDIT scores from AUDIT-10 and AUDIT-P. The models explored the crude and adjusted (for age and sex) associations. Multicollinearity was examined using the variance inflation factor (VIF) and tolerance values. Lagged longitudinal mediation analyses were conducted using Model 4 of the PROCESS macro for SPSS [45] to explore potential indirect associations between rs324420 genotype and AUDIT scores via subjective well-being. We controlled for alcohol use from PART Wave I to remove its potential impact on well-being in the same wave. We also adjusted for potential confounders of the well-being/AUDIT relationship including age, sex, social support, depression, anxiety, stressful life events, and childhood adversities. The indirect effects of rs324420 on AUDIT scores through well-being were estimated using bias-corrected bootstrap confidence intervals based on 5000 bootstrap samples. All statistical analyses were performed using IBM SPSS Statistics v. 27 (IBM Corp, Armonk, NY, USA) with a significance level set at *p* < 0.05.

## 3. Results

### 3.1. Participant and Genotype Characteristics

In the genotyped participants from PART Wave I, the mean age was 44.5 years with a standard deviation (SD) of 12.1, ranging from 19 to 65 years. Additionally, females constituted 59% of this cohort. Table 1 illustrates the sociodemographic and diagnostic attributes of genotyped participants, set against subjective well-being scores from PART Wave I. Younger age, female sex, low social support, and a diagnosis of anxiety or depression were significantly associated with lower well-being scores (*p* < 0.001; Table 1). Similarly, lower well-being was significantly associated with experiences of stressful life events in the prior year or reported childhood adversities (*p* < 0.001; Table 1). The rs324420 genotyping results conformed to the Hardy–Weinberg equilibrium (chi-square = 0.495, *p* = 0.780), with observed genotype frequencies [N = 1713 CC carriers (60.7%), N = 963 AC carriers (34.12%), and N = 146 AA carriers (5.17%)] in line with the overall allele frequencies reported by dbGaP for the total population (C = 0.7946, A = 0.2053, and sample size = 374,708; α Allele Frequency release version: 20201027095038).

### 3.2. The FAAH Activity-Reducing Allele Is Associated with Decreased Subjective Well-Being at Two Distinct Time Points

**Figure 1:**
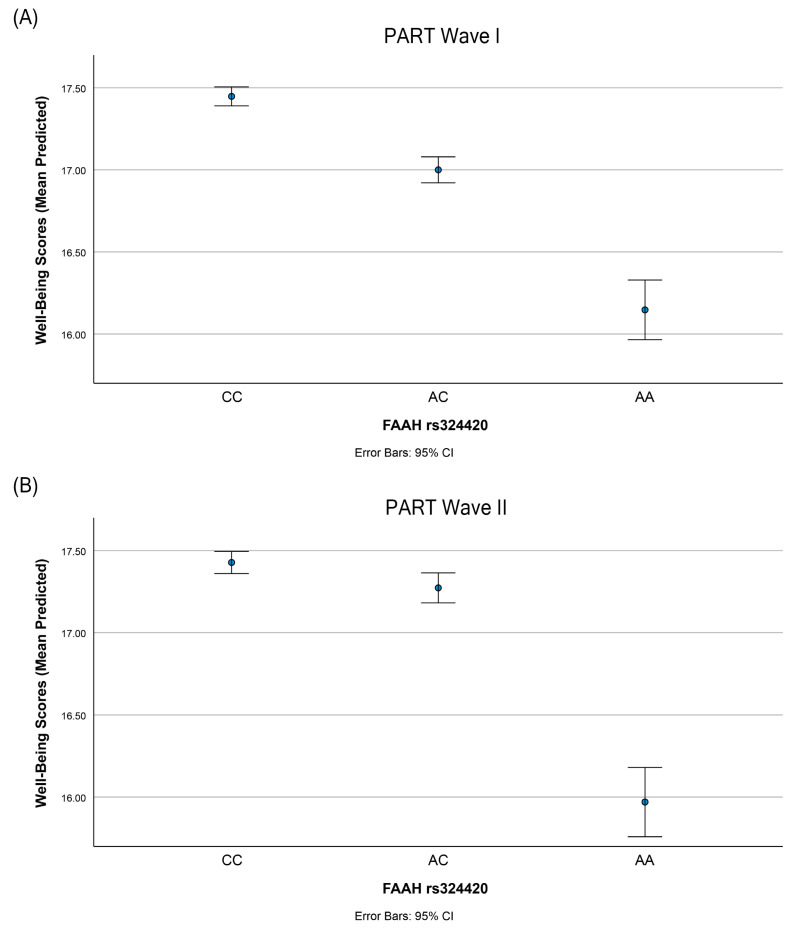
Reduced FAAH activity is associated with lower subjective well-being. The A-allele of rs324420, which encodes threonine, is known to reduce FAAH expression and activity. The linear regression analyses conducted on subjective well-being against rs324420 revealed that well-being scores were significantly lower in A-homozygotes compared to C-homozygotes (Table 3). The graph illustrates the relationship between the three genotype groups of rs324420 and their respective predicted well-being scores in (**A**) PART Wave I and in (**B**) PART Wave II, calculated using coefficients from the regressions adjusted for age and sex. The error bars represent the 95% confidence intervals (CI).

Analyzing data from PART Wave I, we identified a significant dose-dependent association between the number of A-alleles at rs324420 (known to reduce FAAH’s expression and activity) and lower subjective well-being. Each additional A-allele was associated with a decrease in well-being scores of 0.54 units (B: −0.54, *p* < 0.01; Table 2). This association remained significant after adjusting for age and sex (B: −0.52, *p* < 0.01; Table 2). A similar significant association was observed in the PART Wave II data, collected three years after Wave I, in both the crude (B: −0.42, *p* = 0.03; Table 2) and adjusted models (B: −0.41, *p* = 0.03; Table 2). We then evaluated the distinct contributions of the heterozygous (AC) and homozygous risk allele (AA) genotypes to well-being levels, using the homozygous non-risk allele genotype (CC) as the reference. In the PART Wave I data, individuals homozygous for the A-allele exhibited significantly lower well-being in both the crude (B: −1.29, *p* = 0.01; Table 3) and adjusted models (B: −1.17, *p* = 0.02; Table 3). Heterozygous individuals (AC) also demonstrated a trend towards lower well-being, although this trend did not reach significance in the crude model (B: −0.46, *p* = 0.06) and was at the threshold of significance in the adjusted model (B: −0.47, *p* = 0.05). This pattern, with A-allele homozygosity correlating significantly with lower well-being levels but no significant correlation with heterozygosity, was replicated in the PART Wave II data in both crude (AA genotype, B: −1.45, *p* < 0.01; Table 3) and adjusted models (AA genotype, B: −1.36, *p* = 0.01; Table 3). Figure 1 graphically displays these findings for PART Wave I (Figure 1A) and PART Wave II (Figure 1B), presenting mean predicted well-being scores by genotype group, based on the coefficients from the regression adjusted for age and sex.

### 3.3. Affirmation of the Genetic Association between FAAH’s rs324420 and Subjective Well-Being through PheWAS

To corroborate our findings across different cohorts, we performed a phenome-wide association study (PheWAS) for rs324420, encompassing 4756 GWASs and identifying 66 traits significantly linked to rs324420 (*p* < 0.01; Table S1). Within the psychiatric domain, the PheWAS unveiled three significant traits, the first of which affirmed our well-being results: (a) happiness and subjective well-being (Effect allele: C, N = 126,132, *p* = 0.008, Cohort: UK Biobank), (b) nap during day (Effect allele: A, N = 386,124, *p* = 0.00006, Cohort: UK Biobank), and (c) alcohol dependence (Effect allele: A, N = 1161, *p* = 0.005, Cohort: the Study of Addiction, Genetics and Environment; SAGE).

### 3.4. Indirect Association between FAAH’s rs324420 and Problematic Alcohol Use through Lower Subjective Well-Being

The PheWAS results within the psychiatric domain suggested an association between the A-allele of rs324420 and alcohol dependence. To further investigate this relationship, we used AUDIT data from our cohort. However, we did not observe a significant association between rs324420 and AUDIT-10 or AUDIT-P (Table S2). This inconsistency led us to consider the possibility of an indirect relationship between the A-allele of rs324420 and increased alcohol use, potentially facilitated through lower subjective well-being. To explore this hypothesis, we conducted a lagged longitudinal mediation analysis using well-being scores from PART Wave I and AUDIT data from PART Wave II. This analysis showed no direct association between the *FAAH* genotype and the two AUDIT scales but did reveal significant indirect associations via lower well-being (Table 4). However, upon controlling for alcohol use levels from PART Wave I (to account for alcohol’s potential impact on well-being, given the observed negative correlation between AUDIT-10 scores and well-being; Pearson’s r = −0.153, *p* < 0.001) and other potential confounders from PART Wave II, significant indirect associations persisted for AUDIT-P only (Table 4). Taken together, these findings support the hypothesis that decreased FAAH activity may indirectly contribute to problematic alcohol use through its impact on well-being.

## 4. Discussion

Our study revealed a significant dose-dependent association between the minor (A; Thr) allele of the rs324420 genetic variant, a mutation that downregulates the expression and activity of FAAH—the enzyme primarily responsible for breaking down the endocannabinoid anandamide—and lower subjective well-being, as measured by the standardized WHO (Ten) Well-Being Index. This observation held across two measurement periods, three years apart, with each additional A-allele associated with a decrease in well-being scores. Importantly, these findings were not only observed in crude models but also remained significant after adjusting for age and sex, and further affirmation was obtained through a PheWAS analysis using GWAS data from a distinct and substantially larger cohort—the UK Biobank. Our investigation also illuminated a potential connection between rs324420 and alcohol consumption. Specifically, lagged longitudinal mediation analyses revealed significant indirect associations between the *FAAH* genotype and AUDIT scores via lower well-being. However, when controlling for alcohol use levels from PART Wave I and other potential confounders, the indirect associations persisted only for problematic alcohol use, as measured by AUDIT-P. These findings highlight the potential for decreased FAAH activity to indirectly contribute to problematic alcohol use through its impact on well-being.

Interestingly, the only preceding study to associate the *FAAH* gene with well-being utilized population genetic data from an allele frequency database, correlating the allele frequencies of rs324420 with national averages of self-reported happiness [21]. This study identified an inverse association to ours, with the A-allele linked to increased happiness. This contrasting finding is likely attributed to the different methodologies employed; the previous study relied on an allele frequency database, while ours employed direct genotyping of the study group. Our results are further substantiated by replication efforts using a PheWAS, which linked the alternative C-allele with elevated happiness in a GWAS employing the UK Biobank [44]. Additionally, evidence points to the A-allele of rs324420 as a potential risk factor for anxiety and depression [46], which also aligns with our genetic findings. Notably, individuals homozygous for the C-allele of rs324420 have been found to exhibit more positive affective states post placebo administration [47] and increased happiness following cannabis use [48].

While our findings demonstrate a robust association, it is noteworthy that no GWAS to date has linked *FAAH* to well-being at a genome-wide significance level (i.e., *p* < 5 × 10^−8^). Most GWAS studies of well-being have relied on single-item measures or well-being indices not derived from validated instruments, which might have limited their ability to detect this association. Nonetheless, several GWAS studies examining well-being through different measures have succeeded in identifying several genome-wide significant variants [5,6,7,49]. One recent study attempting to leverage all available well-being GWAS data to prioritize and functionally annotate variants of importance identified three genes—*PSMC3*, *ITIH4*, and *SERPINC1*—as probable candidates in the regulation of the well-being spectrum [50]. In light of our findings, which were obtained using the standardized WHO (Ten) Well-Being Index and further supported by a PheWAS analysis, we propose that *FAAH* may also be a significant contributor to well-being. If this is the case, *FAAH* may emerge as a genome-wide significant locus in future larger-scale GWAS studies on well-being, particularly those employing more comprehensive and validated measures of well-being.

In our study, we also found evidence of an indirect association between *FAAH*’s rs324420 and problematic alcohol use, potentially mediated by a decrease in well-being. Prior research has suggested a link between FAAH and various substance use disorders, including alcohol and other drugs [22,24,51,52,53,54,55,56,57], although conflicting findings have been noted regarding alcohol use [58] and rs324420′s effect allele [51]. Our PheWAS analysis corroborated these links, but interestingly, we did not identify a direct association between rs324420 and alcohol use in our cohort. This discrepancy could be due to our study being underpowered. However, the largest GWAS to date on alcohol use phenotypes, including alcohol use disorder [59], problematic alcohol use [60], and drinks per week [61], have also not found genome-wide associations with *FAAH*. This suggested to us that the relationship between *FAAH*’s rs324420 and substance use may be indirect, via reduced well-being that prompts substance use. Indeed, our lagged longitudinal mediation analysis using well-being scores from PART Wave I and AUDIT data from PART Wave II revealed significant indirect associations through well-being. These associations held, specifically for problematic alcohol use (AUDIT-P), even after controlling for potential confounders, including baseline alcohol use. This aligns with previous studies that have suggested the relationship between the rs324420 A-allele and drinking outcomes might be mediated by coping motives [57]. Specifically, the *FAAH*’s rs324420 AC/AA genotype group showed a significant indirect effect indicating a higher propensity to drink to alleviate negative mood or to escape worries [57]. Taken together, these data reinforce the idea that lower FAAH activity might instigate excessive alcohol consumption by impacting psychological aspects of negative reinforcement, a key factor implicated in substance use disorders [62].

Although our study provides robust associations, it is primarily limited by its correlational nature, preventing us from providing direct mechanistic insights into how FAAH’s reduced activity contributes to the observed associations. Preclinical research with *Faah* knockout mice has lent credence to the idea that behavioral changes brought on by increased anandamide levels in the brain mainly transpire through the CB1 receptor [63]. Additionally, studies using CB1 knockouts have suggested a receptor/ligand interplay between CB1 and anandamide, where tonic activation of the receptor seems to govern the biosynthesis of this endogenous ligand [64]. Insights into the effects of FAAH and anandamide could also be drawn from cannabis studies, given that its psychoactive component, tetrahydrocannabinol (THC), also activates the CB1 receptor. Previous human research has linked frequent teenage cannabis use to poorer functional well-being and problematic substance use [65]. Similarly, animal studies have consistently identified prenatal and adolescent neurodevelopmental periods as crucial to understanding the detrimental behavioral and molecular effects of exogenously administered cannabinoids [66,67,68,69,70]. Consequently, we posit that FAAH may play a key role in modulating well-being through its influence on anandamide levels which act on cannabinoid receptors crucial for healthy neurodevelopment [71]. However, it is important to note that FAAH’s enzymatic activity extends beyond anandamide to include a broader array of fatty acid amides, including other N-acylethanolamines [9], oleamide [72], and N-acyltaurines [73]. Anandamide also goes beyond interacting solely with cannabinoid receptors; it serves as a full agonist of the transient receptor potential vanilloid type-1 (TRPV1) ion channel [74] and can activate the nuclear receptor peroxisome proliferator-activated receptor γ (PPARγ) [75]. Given these additional downstream pathways, future investigations into FAAH’s influence on well-being should consider all these potential interactions.

In conclusion, our study provides compelling genetic evidence supporting FAAH’s role in subjective well-being. This association was found to be significant at two distinct time points within the same cohort and was further corroborated in an independent cohort. To the best of our knowledge, we also present the first evidence of a possible indirect association between *FAAH* and alcohol use through well-being. Based on our results, we propose that genetically driven elevation of anandamide levels, inherent in carriers of FAAH’s rs324420 A-allele, might disrupt the endocannabinoid system from conception onwards. This disruption could increase the likelihood of diminished well-being, which in turn could lead to problematic alcohol consumption. However, more genetic studies and mediation analyses are necessary to validate our findings. Furthermore, additional translational and mechanistic investigations are required to comprehend the role of cannabinoid compounds and their target receptors in shaping complex psychological constructs such as well-being.

## Figures and Tables

**Table 1:** Well-being scores across sociodemographic and diagnostic characteristics of the genotyped study group.

Characteristics	Groups	N	Well-Being Score Mean (Median)
Age group *	19–24	169	16.3 (17)
	25–34	531	16.5 (17)
	35–44	582	16.1 (17)
	45–54	796	17.2 (18)
	55–65	706	18.8 (19)
			
Sex *	Male	1142	18.3 (19)
	Female	1643	16.4 (17)
			
Education	Primary School	403	16.9 (18)
	Secondary School	1046	17.0 (17)
	Tertiary Education	1304	17.4 (18)
			
Social support *	Low	711	14.1 (14)
	High	2068	18.3 (19)
			
Diagnosis *	No anxiety or depression	2187	18.9 (19)
	Anxiety	160	14.0 (13)
	Depression	436	9.9 (10)
			
Stressful life events *	No	780	19.2 (19)
	Yes	1992	16.4 (17)
			
Childhood adversities *	No	1997	17.7 (18)
	Yes	656	15.6 (16)

* Statistically significant differences on well-being scores according to Kruskal–Wallis test (*p* < 0.001).

**Table 2:** Linear regressions of subjective well-being scores on FAAH genotype ^‡^.

PART Wave	N	B (95% CI) ^a^	*p*	N	B (95% CI) ^b^	*p*
Wave I	2785	−0.54 (−0.93, −0.16)	0.006	2784	−0.52 (−0.90, −0.14)	0.007
						
Wave II	2777	−0.42 (−0.82, −0.03)	0.03	2776	−0.41 (−0.79, −0.02)	0.03

^‡^ rs324420: CC (coded 0), AC (coded 1), and AA (coded 2); ^a^ crude regression; ^b^ adjusted for age and sex; B, unstandardized β coefficient, CI, confidence Interval, and *p*, *p*-value.

**Table 3:** Linear regressions of subjective well-being scores on individual risk-allele FAAH genotypes ^‡^.

PART Wave	rs324420	N	B (95% CI) ^a^	*p*	N	B (95% CI) ^b^	*p*
Wave I	AC	2785	−0.46 (−0.95, 0.02)	0.06	2784	−0.47 (−0.95, 0.01)	0.05
	AA		−1.29 (−2.35, −0.24)	0.01		−1.17 (−2.20, −0.14)	0.02
							
Wave II	AC	2777	−0.17 (−0.67, 0.33)	0.50	2776	−0.17 (−0.66, 0.31)	0.47
	AA		−1.45 (−2.53, −0.38)	0.008		−1.36 (−2.41, −0.31)	0.01

^‡^ rs324420: AC or AA compared to CC; ^a^ crude regression; ^b^ adjusted for age and sex; B, unstandardized β coefficient, CI, confidence Interval, and *p*, *p*-value.

**Table 4:** Mediation analyses of the FAAH ^‡^ genotype’s effect on alcohol use through well-being.

AUDIT Scale	N	Direct Effect (Boot 95% CI)	Sig	Indirect Effect (Boot 95% CI)	Sig
AUDIT-10 ^a^	2622	−0.096 (−0.285, 0.092)	No	0.033 (0.007, 0.064)	Yes
AUDIT-10 ^b^	2550	−0.082 (−0.212, 0.047)	No	0.004 (−0.002, 0.015)	No
AUDIT-10 ^c^	2411	−0.044 (−0.179, 0.091)	No	0.002 (−0.003, 0.011)	No
					
AUDIT-P ^a^	2628	0.006 (−0.107, 0.119)	No	0.030 (0.007, 0.056)	Yes
AUDIT-P ^b^	2555	0.019 (−0.071, 0.110)	No	0.015 (0.003, 0.030)	Yes
AUDIT-P ^c^	2416	0.036 (−0.058, 0.131)	No	0.005 (0.0003, 0.012)	Yes

^‡^ rs324420: CC (coded 0), AC (coded 1), and AA (coded 2); ^a^ crude model; ^b^ model adjusted for AUDIT-10 in PART Wave I; ^c^ model adjusted for AUDIT-10 in PART Wave I, age, sex, and social support in PART Wave II, depression, anxiety, and stressful life events in PART Wave II, and childhood adversities; AUDIT-10: full AUDIT scale (10 items), AUDIT-P: alcohol problem items; Boot CI: Bootstrap Confidence Interval; Sig: Statistical significance.

## Data Availability

The data presented in this study are available on request from the corresponding author. The data are not publicly available due to ethical and privacy restrictions.

## Supplementary Materials

The following supporting information can be downloaded at: https://www.mdpi.com/article/10.3390/genes14091826/s1, Table S1: Phenome-Wide Association Study (PheWAS) identified 66 traits significantly linked to *FAAH*’s rs324420 at *p* < 0.01. Within the psychiatric domain, the PheWAS unveiled three significant traits (highlighted in yellow); Table S2: Linear regression of alcohol use on *FAAH* genotype.
