# Supplementary material for: Functional Variation in the FAAH Gene Is Directly Associated with Subjective Well-Being and Indirectly Associated with Problematic Alcohol Use
Source: Genes (Basel). 2023 Sep 21;14(9):1826. doi: 10.3390/genes14091826 (PMC10530831; doi:10.3390/genes14091826)
Supplement: Supplementary file 1 [file genes-14-01826-s001.zip › genes-2620053-supplementary.pdf]

Supplementary material

Table S1. Phenome-Wide Association Study (PheWAS) identified 66 traits significantly linked to *FAAH*'s rs324420 at p<0.01. Within the psychiatric domain, the PheWAS unveiled three significant traits (highlighted in yellow).

Domain	Trait	P-value	N	EA
Activities	Number of days/week of vigorous physical activity 10+ minutes	0.003794	368164	C
Activities	Time spent driving	0.005381	258336	A
Activities	Types of physical activity in last 4 weeks: Other exercises (eg: swimming, cycling, keep fit, bowling)	0.001304	384450	C
Activities	Positive affect (univariate)	0.009076533	410603	A
Activities	Strenuous sports or other exercises	0.0047	350492	C
Activities	Vigorous physical activity	0.0066	261055	C
Activities	Moderate to vigorous physical activity levels	0.00074	377234	C
Cardiovascular	Heart rate	0.002564	85787	A
Cardiovascular	Pulse rate (automated reading)	0.0006695	361411	A
Cardiovascular	Heart rate recovery at 40 secnds	0.0069	58818	C
Cardiovascular	Posterior wall thickness	0.009263	19373	A
Cardiovascular	Left ventricular mass	0.003373	19076	A
Cardiovascular	Resting heart rate	0.0000085	458969	A
Dermatological	Male-specific factors - Hair/balding pattern: Pattern 1	0.001682	176380	C
Dermatological	Male pattern baldness (BOLT LMM infinitesimal mixed model)	0.0072	205327	A
Dermatological	Male pattern baldness (BOLT LMM non-infinitesimal mixed model)	0.0087	205327	A
Environment	Illnesses of mother: Breast cancer	0.006447	367939	C
Gastrointestinal	Ulcerative colitis	0.005761	45975	A
Gastrointestinal	Non-cancer illness code, self-reported: gastro-oesophageal reflux (gord) / gastric reflux	0.002621	289307	A
Immunological	CD123 on 11c+123+DC	0.004897054	669	A
Metabolic	Amino acid::Glycine, serine and threonine metabolism::threonine	0.009639	6020	A
Metabolic	Amino acid::Histidine metabolism::histidine	0.00237	7804	A
Metabolic	Lipid::Bile acid metabolism::glycochenodeoxycholate	0.003626	7087	A
Metabolic	Lipid::Carnitine metabolism::carnitine	0.009458	7797	A
Metabolic	Lipid::Essential fatty acid::docosahexaenoate (DHA; 22:6n3)	0.001206	7818	C
Metabolic	Lipid::Essential fatty acid::linolenate [alpha or gamma; (18:3n3 or 6)]	0.006324	7786	C

Metabolic	Lipid::Fatty acid metabolism (also BCAA metabolism)::propionylcarnitine	0.002213	7813	A
Metabolic	Lipid::Long chain fatty acid::stearidonate (18:4n3)	0.001075	7775	C
Metabolic	Lipid::Lysolipid::1-arachidonoylglycerophosphoethanolamine*	0.00762	7798	A
Metabolic	Lipid::Lysolipid::1-heptadecanoylglycerophosphocholine	0.006804	7422	C
Metabolic	Peptide::gamma-glutamyl::gamma-glutamylvaline	0.0009626	7753	A
Metabolic	::::X-06126	0.005869	7785	A
Metabolic	::::X-11261	0.004773	7771	C
Metabolic	::::X-11374	0.001753	2609	A
Metabolic	::::X-11478	0.008187	6593	C
Metabolic	::::X-11529	0.005358	6664	A
Metabolic	::::X-11792	0.002144	2442	C
Metabolic	Peptide::Dipeptide::X-12244--N-acetylcarnosine	0.001479	6608	C
Metabolic	::::X-12556	0.006334	7483	A
Metabolic	::::X-12729	0.006026	1753	C
Metabolic	22:6, docosahexaenoic acid (DHA)	0.008215	13499	C
Metabolic	OmegaL3 fatty acids	0.001872	13544	C
Metabolic	Adiponectin	0.004142	7825	C
Metabolic	25-Hydroxyvitamin D level	0.008131	79366	C
Neurological	Inferior fronto-occipital fasciculus fractional anisotropy	0.001681	17706	A
Neurological	Splenium of corpus callosum fractional anisotropy	0.008783	17706	A
Neurological	External capsule mean diusivities	0.009321	17706	C
Neurological	Inferior fronto-occipital fasciculus mean diusivities	0.001051	17706	C
Neurological	External capsule radial diusivities	0.009843	17706	C
Neurological	Inferior fronto-occipital fasciculus radial diusivities	0.0001234	17706	C
Nutritional	Lamb/mutton intake	0.009147	384188	A
Nutritional	Bread intake	0.002949	377627	A
Nutritional	Never eat eggs, dairy, wheat, sugar: Sugar or foods/drinks containing sugar	0.002323	384986	C
Nutritional	Never eat eggs, dairy, wheat, sugar: I eat all of the above	0.001757	384986	A
Psychiatric	Alcohol dependence	0.0050206	1161	A
Psychiatric	Nap during day	0.00006174	386124	A
Psychiatric	Happiness and subjective well-being - General happiness	0.008221	126132	C
Reproduction	Had menopause (female)	0.009168	175519	A
Reproduction	Dysmenorrhea pain severity	0.006661616	11348	C

Reproduction	Menstruation quality of life impact: Increased appetite	0.004765989	11348	C
Skeletal	Total-body less head BMD	0.00412804	10414	A
Skeletal	Total-body lean mass	0.0092822	10414	A
Skeletal	Total-body less head BMD and total body lean mass (bivariate meta-analysis)	0.00515213	10414	NA
Skeletal	Osteoarthritis of hip or knee (hospital diagnosed)	0.00577852	32970	C
Skeletal	Heel bone mineral density	0.0056	394929	C
Social Interactions	Number of full brothers	0.00537	380062	A

Table S2. Linear regression of alcohol use on *FAAH* genotype[‡]

PART Wave	AUDIT scale	N	B (95% CI)[a]	P	B (95% CI)[b]	P
Wave I	AUDIT-10	2,647	0.02 (-0.16, 0.21)	0.78	0.07 (-0.11, 0.25)	0.45
	AUDIT-P	2,653	0.04 (-0.06, 0.16)	0.42	0.05 (-0.05, 0.17)	0.30
Wave II	AUDIT-10	2,644	-0.07 (-0.26, 0.11)	0.45	-0.03 (-0.21, 0.15)	0.72
	AUDIT-P	2,651	0.02 (-0.08, 0.14)	0.64	0.04 (-0.07, 0.15)	0.48

[‡] rs324420: CC (coded 0), AC (coded 1), AA (coded 2)

[a] Crude regression

[b] Adjusted for age and sex
